# Per2 attenuates LPS-induced chondrocyte injury through the PTEN/PI3K/Akt signalling pathway

**DOI:** 10.1042/BSR20200417

**Published:** 2020-05-28

**Authors:** Yu Zhao, Ding Ma, Bingchen Dong, Ming Li

**Affiliations:** 1 Depatment of Orthopaedics, Xi’an Ninth Hospital, Xi'an 710054, Shaanxi Province, PR China; 2 Depatment of Geriatrics, Xi’an Ninth Hospital, Xi’an 710054, Shaanxi Province, PR China

**Keywords:** chondrocyte, inflammation, osteoarthritis, Per2, PTEN/PI3K/Akt

## Abstract

This research aimed to explore the role of period circadian clock 2 (Per2) in the evolution of osteoarthritis (OA) and the relevant mechanisms. Per2 messenger RNA (mRNA) and protein levels were markedly reduced in NHAC-kn cells treated with 5 µg/ml lipopolysaccharide (LPS) for 12 h. Then, pcDNA3.1-Per2 and si-Per2 were recruited to boost and reduce the expression of Per2, respectively. MTT assay, apoptosis analysis and enzyme-linked immunosorbent assay (ELISA) results showed that Per2 increased cell proliferation, while inhibited apoptosis and inflammation. Furthermore, the PTEN/PI3K/Akt signalling pathway was activated by Per2 overexpression; the CO-IP data confirmed that Per2 specifically bound to PTEN. Through employing IGF-1, a PI3K activator, we determined that Per2-mediated inflammation response in LPS-stimulated NHAC-kn cells through the PTEN/PI3K/Akt signalling pathway. In summary, the present study indicates that Per2 may serve as a novel therapeutic target through activating the PTEN/PI3K/Akt signalling pathway.

## Introduction

Osteoarthritis (OA), a kind of chronic degenerative joint disease, is prevalent among the elderly. With the destruction of articular cartilage and bone proliferation around joints as the main pathological features, OA is manifested as joint pain, joint stiffness, loss of mobility and disability. OA is mainly related to age, gender and genetic factors [1–4]. Except for joint replacement, there are few available therapies for OA; thus, exploring novel therapeutic targets is imperative.

Circadian genes are imperative regulators of immune function [5]. Existing research has demonstrated that circadian clock genes influence some downstream genes in cartilage tissue and, in turn, participate in related diseases [6]. Furthermore, these genes also affect endochondral ossification, and there is circadian variation in chondrocyte proliferation and the change rate of growth plates [7]. Period circadian clock 2 (Per2) participates in inhibiting tumours via targeting other genes that are relevant to cell proliferation and death, including cyclin A1, transformed mouse 3T3 cell double minute 2 and cyclin-dependent kinase 1 [8,9]. In addition, by controlling the rhythmic secretion of glucocorticoids (GCs) and gating the responses of mast cells to GCs at certain times of day, Per2 regulates time-dependent changes of passive skin allergy in mice [10]. Morayo et al. demonstrated that Per2 curbs pulmonary inflammation in sickle cell disease [11]. Furthermore, RNA sequencing analysis showed that Per2 is a potential target in temporomandibular joint OA treatment in rats [12]. Per2 knockdown evidently improves proliferation and migration while inhibiting apoptosis of MNNG/HOS osteosarcoma cells [13]. However, the effects of Per2 on the development of OA are not yet clear.

Phosphatase and tensin homologue deleted on chromosome 10 (PTEN) is viewed as a critical tumour suppressor for glioblastoma, prostate cancer and breast cancer. Besides, PTEN reportedly participates in multiple cellular processes, such as proliferation, cellular architecture and survival [14]. A previous study demonstrated that PTEN may also play a key role in inflammatory responses [15]. Agrawal et al. reported that overexpressing PTEN in dendritic cells from elderly individuals markedly increases lipopolysaccharide (LPS)-induced secretion of tumour necrosis factor (TNF)-α and interleukin (IL)-6 [16]. Recently, PTEN has been reported to participate in OA initiation. Specifically, PTEN expression is increased in osteoarthritic human cartilage and chondrocytes and is associated with chondrocyte responses and matrix synthesis [17]. Furthermore, PTEN negatively regulates phosphoinositide 3-kinase/protein kinase B (PI3K/AKT) signalling pathway [18], which is a vital factor in OA chondrocytes proliferation and differentiation [19].

Our study aimed to explore the effects of Per2 on the proliferation, apoptosis and inflammation of OA. To investigate the mechanism of Per2 in OA, we also evaluated the relationship between Per2 and the PTEN/PI3k/Akt signalling pathway.

## Materials and methods

### Cell culture, treatment and transfection

NHAC-kn cells (Cambrex, Walkersville, MD) were cultured in Dulbecco’s Modified Eagle’s Medium (DMEM; HyClone) with 10% foetal bovine serum (HyClone). NHAC-kn cells were seeded in 24-well plates with a cell density of 1 × 10^5^ per well to establish an OA cell model. NHAC-kn cells were grown to 80% confluency, followed by LPS treatment (Sigma-Aldrich; Merck KGaA, Darmstadt, Germany) at 0, 1, 5 or 10 µg/ml for 12 h. Cells without treatment were defined as untreated controls.

To overexpress and inhibit Per2 expression, pcDNA3.1-Per2 and si-Per2, respectively, were transfected into NHAC-kn cells, followed by culturing for 24 h. And insulin-like growth factor-1 (IGF-1; 10 nM, PI3K activator) was added to cells for 48 h.

### Quantitative real-time polymerase chain reaction (PCR)

Total RNA was obtained from cell samples using the RNeasy Plus Mini Kit (Qiagen). Complementary DNA (cDNA) was synthesised using reverse transcription reagents (ABI, CA). The primer sequences were as follows: Per2 forward: 5′-TCTCCCTAGTGATGCGCTTG-3′ and reverse: 5′-CAGCAGCCCAAGGAACTT-3′; si-Per2: 5′-GCGCUAAGGUCCAGUGAUA-3′ and 5′-UAUCACUGGACCUUAGCGC-3′; GAPDH forward: 5′-ACTTTGGTATCGTGGAAGGACTCAT-3′ and reverse: 5′-GTTTTTCTAGACGGCAGGTCAGG-3′. pcDNA3.1-Per2 primers were designed and purchased from Invitrogen. Quantitative real-time PCR was implemented using a Bio-Rad iCycler IQ RealTime PCR Detection System (Bio-Rad Laboratories). The 2^−ΔΔCt^ method was employed to normalise Per2 expression.

### MTT assay

Cells were cultured for 0, 12, 24 h and then 10 µl MTT (0.5 mg/ml; Invitrogen) was added; the cells were incubated at 37°C for 4 h. Subsequently, the culture medium was aspirated and 150 µl dimethyl sulfoxide (DMSO; Invitrogen) was added to each well the plate was incubated for 15 min. The absorbance at 570 nm was then measured.

### Apoptosis assay

An Annexin V-FITC/PI kit (BD, U.S.A.) was employed to evaluate the cell apoptotic. Cells were washed and resuspended (1 × 10^5^ cells/ml) and then stained with Annexin V-FITC/PI (Invitrogen) in the dark for 15 min. The apoptosis rate was measured with FlowJo software.

### Enzyme-linked immunosorbent assay (ELISA)

Cells were centrifuged, and the pellet was disrupted in radioimmunoprecipitation assay (RIPA) buffer for 15 min. The protein concentration in the extract was evaluated using the BCA Protein Assay kit (Pierce). The protein was centrifuged at 1000 ***g*** for 10 min, and the levels of interleukin (IL)-6, IL-8 and tumour necrosis factor alpha (TNF-α) were determined by ELISA kits (Nanjing Jiancheng Bioengineering Research Institute).

### Co-immunoprecipitation (Co-IP)

Protein was collected from cells by RIPA buffer. Four micrograms of primary antibody was added to 1000 μg protein and incubated for 8 h at 4°C. Then, protein A Sepharose beads (Santa Cruz, Texas, U.S.A.) were added. After 1 h incubation at 4°C, the beads were centrifuged at 800 ***g*** for 3 min and resuspended with 5 × loading buffer. Finally, Western blot was employed to analyse the protein sample.

### Western blotting

Cells were lysed in lysis buffer and qualified using the BCA Protein Assay kit. Fifty micrograms of protein samples were subjected to sodium dodecyl sulfate-polyacrylamide gel electrophoresis (SDS-PAGE) electrophoresis. The separated proteins were transferred to polyvinylidene fluoride (PVDF) membranes (Invitrogen). Then, PVDF membranes were incubated with 5% nonfat milk in Tris-buffered saline with Tween 20 (TBST) to block nonspecific protein binding, followed by incubation with primary antibody against PTEN (1:500), PI3K (1:500), p-Akt (1:500), p65 (1:500) or glyceraldehyde 3-phosphate dehydrogenase (GAPDH; 1:2000). After three washes in TBST, membranes were incubated with secondary anti-rabbit antibodies conjugated to horseradish peroxidase (1:5000; Cell Signaling Technology, Inc., #7074). The bands were visualised using an enhanced chemiluminescence kit and analysed by AlphaEaseFC 4.0 software.

### Statistical analysis

Statistical analysis was performed via SPSS19.0 software (IBM, U.S.A.). The results are presented as the mean ± standard deviation (SD). The *t*-test and one-way analysis of variance (ANOVA) were utilized to compare the differences between two and more than two groups, respectively. *P*<0.05 difference was considered statistically significant.

## Results

### PER2 expression was low in LPS-induced NHAC-kn cells

**Figure 1:**
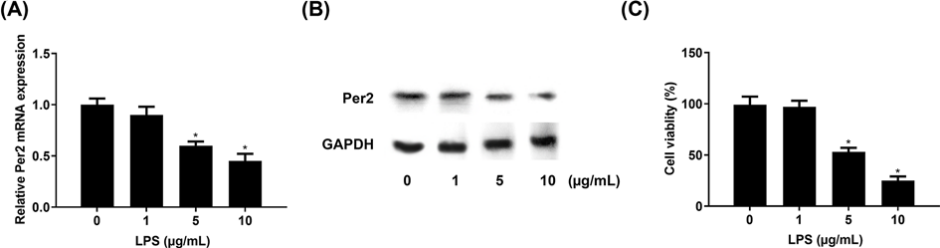
The Per2 expression level was decreased in lipopolysaccharide (LPS)-treated NHAC-kn cells Cells were cultured and treated with 0, 1, 5 or 10 μg/ml LPS for 12 h. Per2 (**A**) messenger RNA (mRNA) and (**B**) protein levels in LPS-treated NHAC-kn cells were explored by quantitative real-time polymerase chain reaction and Western blot, respectively. (**C**) Cell viability was evaluated using the MTT assay. *P<0.05 vs. no LPS treatment.

Per2 mRNA and protein expression were significantly decreased in NHAC-kn cells after 5 and 10 μg/ml LPS treatment for 12 h compared with untreated cells (Figure 1A,B). The cell viability was decreased by LPS stimulation, which meant that the OA cell model was built successfully (Figure 1C). The NHAC-kn cells treated with 5 μg/ml LPS for 12 h was used in the subsequent experiment.

### Per2 increased NHAC-kn cell proliferation and decreased apoptosis

**Figure 2:**
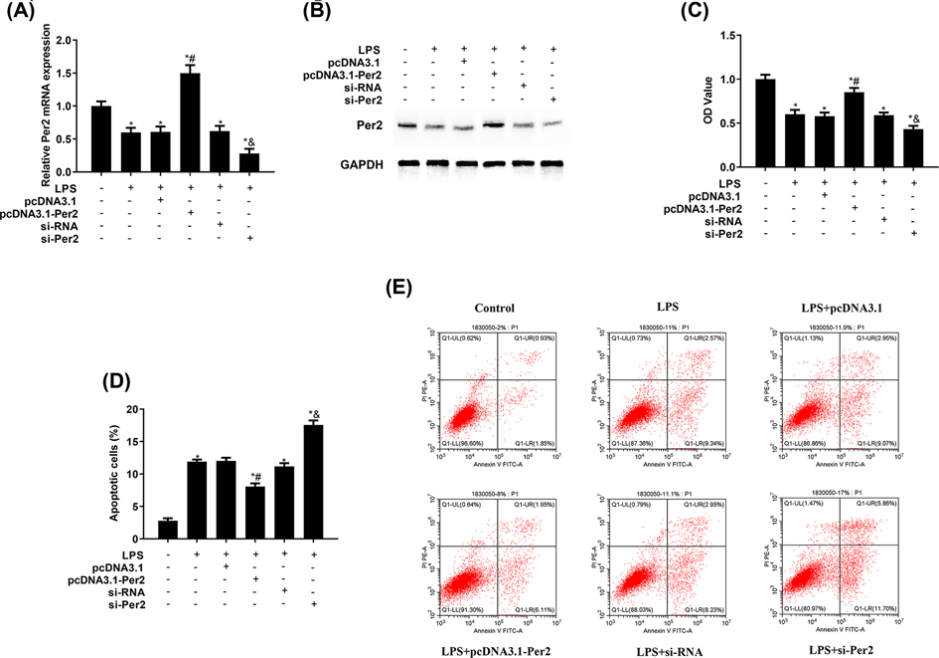
Per2 increased cell proliferation and decreased apoptosis in NHAC-kn cells NHAC-kn cells transfected with pcDNA3.1-Per2 or si-Per2 were treated with 5 μg/ml lipopolysaccharide (LPS) for 12 h. Per2 (**A**) messenger RNA (mRNA) and (**B**) protein expression were evaluated via quantitative real-time polymerase chain reaction and Western blot, respectively. (**C**) Cell proliferation and (**D** and **E**) apoptosis were analysed via the MTT and an apoptosis assay, respectively. *P<0.05 vs. no LPS treatment; ^#^*P*<0.05 vs. pcDNA3.1; ^&^*P*<0.05 vs. si-RNA.

To explore the role of Per2 in cell proliferation and apoptosis, pcDNA3.1-Per2 and si-Per2 were used to enhance and reduce, respectively, the expression of Per2 (Figure 2A,B). As shown in Figure 2C–E, overexpressing Per2 increased cell proliferation but inhibited cell apoptosis, compared with the pcDNA3.1 group. The si-Per2 group showed the opposite effects. Overall, Per2 was conducive to the growth of LPS-treated NHAC-kn cells.

### Per2 curbed inflammation in NHAC-kn cells

**Figure 3:**
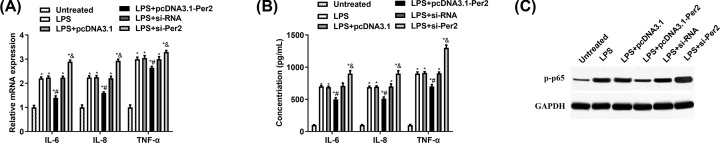
Per2 suppressed inflammation of NHAC-kn cells NHAC-kn cells transfected with pcDNA3.1-Per2 and si-Per2 were treated with 5 μg/ml lipopolysaccharide (LPS) for 12 h. (**A**) Interleukin (IL)-6, IL-8 and tumour necrosis factor alpha (TNF-α) messenger RNA (mRNA) expression were evaluated with quantitative real-time polymerase chain reaction. (**B**) The protein level of IL-6, IL-8 and TNF-α in culture supernatants were quantified by enzyme-linked immunosorbent assay (ELISA). (**C**) The phosphorylation level of NF-κB p65 was determined by Western blot. *P<0.05 vs. no LPS treatment; ^#^*P*<0.05 vs. pcDNA3.1; ^&^*P*<0.05 vs. si-RNA.

To further explore the function of Per2 in the inflammatory response during OA development, its effect on the release of pro-inflammatory cytokines, including IL-6, IL-8 and TNF-α – all of which contribute to the clinical symptoms of OA-was further analysed. IL-6, IL-8 and TNF-α mRNA levels were decreased by overexpressing Per2, while they were elevated by down-regulating Per2 in NHAC-kn cells, compared with their respective control (Figure 3A). ELISA assays revealed that the protein expression matched the mRNA levels (Figure 3B). What’s more, p-p65 level was also down-regulated by overexpressing Per2 (Figure 3C).

### The PTEN/PI3K/Akt signalling pathway participated in the effects of Per2 on OA inflammation response

**Figure 4:**
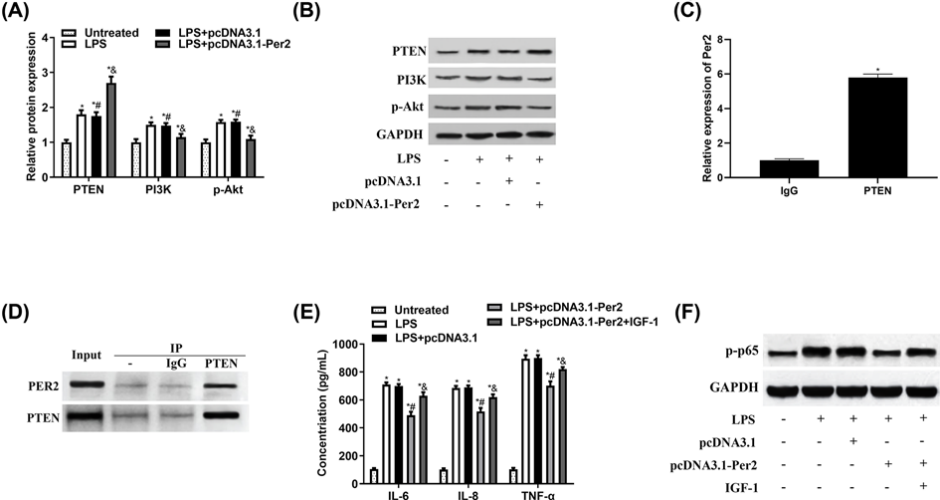
Per2 attenuated inflammation response of LPS-treated NHAC-kn cells by PTEN/PI3K/Akt signalling pathway NHAC-kn cells transfected with pcDNA3.1-Per2 were treated with 5 μg/mL lipopolysaccharide (LPS) for 12 h and then 10 nM insulin-like growth factor-1 (IGF-1). (**A,B**) PTEN, PI3K and Akt protein expression were assessed using Western blot. (**C** and **D**) CO-IP assay was recruited to evaluate interactive relationship between Per2 and PTEN. (**E**) Interleukin (IL)-6, IL-8 and tumour necrosis factor alpha (TNF-α) protein levels in culture supernatants were evaluated by enzyme-linked immunosorbent assay (ELISA). (**F**) p-p65 level was determined by Western blot. *P<0.05 vs. no LPS treatment; ^#^*P*<0.05 vs. pcDNA3.1; ^&^*P*<0.05 vs. pcDNA3.1-Per2.

Per2 and PTEN expression are reportedly positively correlated [20]. Considering that PTEN inhibits PI3K/Akt pathway activation, which promotes inflammation, we explored whether Per2 influenced OA development via PTEN and then PI3K/Akt signalling pathways. As shown in Figure 4A,B, LPS evaluated the expression of PTEN, PI3K and p-Akt. pcDNA3.1-Per2 continued to increase PTEN level, while inhibited PI3K and p-Akt expression.

We next assessed whether PTEN/PI3K/Akt participated in the effects of Per2 in OA. First, it was confirmed that Per2 interacted with PTEN by CO-IP assay (Figure 4C,D). We then analysed the participation of PTEN in the Per2-mediated effects on inflammation divisors of OA and the PI3K activator, IGF-1 was recruited. And it was demonstrated that PI3K activation markedly increased IL-6, IL-8 and TNF-α levels in NHAC-kn cells after Per2 up-regulation (Figure 4E). Furthermore, IGF-1 also had the ability of reversing pcDNA3.1-Per2 effect on p-p65 (Figure 4F). In summary, the PI3K/Akt signalling pathway participated in the effects of Per2 on inflammation in OA.

## Discussion

Joint cartilage, ligaments, subchondral bone, capsule and adjacent muscles are vulnerable to OA [21,22]. OA has the characteristics of chronic joint cartilage degradation and articular fringe as well as subchondral bone sclerosis of the hands, elbows, knees and hips [23,24]. The proliferation and apoptosis of normal articular chondrocytes are in a certain state of equilibrium that ensures the number of articular chondrocytes. Apoptosis of Chondrocyte are more serious in human or animal with OA than the healthy one [25]. OA harms the patients’ quality of life and also causes economic burdens on society [26].

Pervious research has shown that circadian clock genes are prominent factors in the development of inflammation. For example, overexpressing cryptochrome 1 helps moderate vascular inflammation induced by sleep deprivation in mouse models [27]. Noxious stimuli curb BMAL1 expression and then boost a pro-inflammatory environment [28]. Consistently, some circadian clock genes are deregulated in the pathology of joint inflammatory diseases [29]. Per2, a circadian clock gene, is pivotal in resetting the clock. Per2 mutations are related to early vascular senescence, limb ischaemia and endothelial dysfunction [30]. Per2 is also related to inflammation response, like myocardial inflammation [31] and dermatitis [32]. Previous research has demonstrated that Per2 expression was reduced in non-tumourigenic breast epithelial cells stimulated by IL-6 [33]. Further, immunoblotting analysis showed the Per2 protein expression was inhibited in LPS-treated rheumatoid arthritis synovial cells [34]. In the present study, Per2 mRNA expression was markedly lower in NHAC-kn cells treated by LPS than normal NHAC-kn cells. A previous study showed a significant decrease in the proliferation of bone marrow progenitor cells from Per2 mutant mice [30]. Down-regulating Per2 reportedly increases E2F1 expression and curbs p21 expression, which are the dual-directional regulators of apoptosis [35]; this change would lead to a more serious imbalance of cell proliferation and apoptosis [36]. MTT analysis revealed that overexpressing Per2 also induced NHAC-kn cell proliferation while curbing apoptosis after LPS treatment. Proinflammatory cytokines are increased in Per2 mutant mice, and Per2^−/-^ mice show very strong activation of the inflammatory response [37]. Further research in the present study has validated that overexpressing Per2 significantly inhibits the inflammatory response induced by LPS in NHAC-kn cells. NF-κB signaling pathway was reported to be key in regulating inflammatory mediators involved in the OA pathogenesis [38]. And we found that overexpressing Per2 decreased the phosphorylation of NF-κB p65 while si-Per2 activated it. Thus, we hypothesise that Per2 upregulation in cartilage tissues is helpful for OA alleviation.

PTEN expression is reportedly boosted in human osteoarthritic cartilage and chondrocytes, a phenomenon associated with chondrocyte responses and matrix synthesis [39,17]. In our research, there was a similar induction of PTEN expression in pcDNA3.1-transfected OA cell model that led to a decrease of phosphorylated PI3K and AKT. Chen et al. [40] demonstrated that Per2 knockdown resulted in the PI3K/AKT/mTOR signalling pathway activation. In oral cancer, the expression of Per2 reportedly has a positive correlation with PTEN [20]. Hence, in the present study, we attempted to determine whether there was a connecting relationship between Per2 and PTEN. Through CO-IP assay, we confirmed that Per2 interacted with PTEN. Notably, PTEN can inhibit matrix synthesis of OA chondrocytes via the PI3K/AKT signalling pathway [17]. In addition to matrix synthesis, the PI3K/AKT signalling pathway is also a vital regulator of OA chondrocyte proliferation and differentiation. Activating the PI3K/AKT signalling pathway is helpful for the proliferation increase in OA chondrocytes [19]. In the present study, through evaluate the levels of IL-6, IL-8, TNF-α and p-p65, the PI3K activator, IGF-1 was proven to boost the inflammatory response, which was inhibited by pcDNA3.1-Per2 in LPS-treated NHAC-kn cells. Thus, we further confirmed that Per2 regulated the inflammation response of NHAC-kn cells treated with LPS by PTEN/PI3K/AKT signalling pathway.

In summary, we determined that Per2 targeted PTEN/PI3K/Akt signalling to regulate OA development. These findings should prove helpful for gaining a better understanding of the underlying mechanism of OA establishment and development.

## Abbreviations

ELISA: enzyme-linked immunosorbent assay
IL: interleukin
LPS: lipopolysaccharide
OA: osteoarthritis
mRNA: messenger RNA
Per2: period circadian clock 2
PI3K/AKT: phosphatidylinositol 3-kinase/protein kinase B
PTEN: phosphatase and tensin homologue deleted on chromosome 10
TNF: tumour necrosis factor
